# The effect of zinc supplementation on anthropometric measurements in healthy children over two years: a systematic review and meta-analysis

**DOI:** 10.1186/s12887-023-04249-x

**Published:** 2023-08-23

**Authors:** Vahid Monfared, Adel Salehian, Zeinab Nikniaz, Soraiya Ebrahimpour-Koujan, Zeinab Faghfoori

**Affiliations:** 1 Student Research Committee, Semnan University of Medical Sciences, Semnan, Iran; 2 Liver and gastrointestinal diseases research center, Tabriz University of medical sciences, Tabriz, Iran; 3 Department of Clinical Nutrition, School of Nutritional Sciences and Dietetics, Tehran University of Medical Sciences, Tehran, Iran; 4 Food Safety Research Center (salt), Semnan University of Medical Sciences, Semnan, ZIP Code: 3581793563 Iran

**Keywords:** Body height, Body weight, Meta-analysis, Zinc

## Abstract

**Background:**

Zinc deficiency is one of the most important micronutrient deficiencies in children that can affect the children’s growth pattern. In this regard, different studies were conducted to assess the effect of zinc supplementation on growth patterns in healthy children. To the best of our knowledge, no systematic review has summarized the results of these studies. So, in the present study, we systematically reviewed the result of the studies that assessed the effect of zinc supplementation on anthropometric parameters in healthy, over 2-year-old children.

**Methods:**

A systematic search was carried out in PubMed, Scopus, and Web of Science from inception to November 2021. Data were pooled using the random-effects method and were expressed as weighted mean difference (WMD) and 95% confidence intervals (CI).

**Results:**

The pooled results of eight studies, including 1586 participants, showed that zinc supplementation significantly increases height [(WMD): 0.9, 95% CI: (0.27, 1.52), *p* < *0.001*], weight [(WMD): 0.51, 95% CI: (0.06, 0.97), *p* < *0.001*], height for age (HAZ) [(WMD): 0.07, 95% CI: (0.03, 0.10), *p < 0.001*]. Also, meta-regression analysis did not reveal any significant association between dose and duration of intervention and anthropometric parameters.

**Conclusion:**

The present study demonstrates the beneficial effects of zinc supplementation on weight, height, and HAZ.

**Supplementary Information:**

The online version contains supplementary material available at 10.1186/s12887-023-04249-x.

## Background

Child growth is a vital indicator of health status in the human life cycle. Different factors including childhood infections, child undernutrition, and food insecurity could affect the child’s growth [1]. Child undernutrition is the underlying cause of three million annual deaths worldwide [2]. It includes stunting, wasting, and essential micronutrient deficiencies. Failure to properly treat nutritional deficiencies, or even subclinical malnutrition, can have detrimental effects on an individual’s health and the nation’s overall economic growth [3].

One of the most important micronutrient deficiencies especially in children is attributed to zinc deficiency [4]. Zinc insufficiency is a huge issue in terms of public health that affects over two billion people all over the world [5, 6]. In Iran, it has been estimated that 10% of people suffer from zinc deficiency [7, 8]. It has been demonstrated that zinc deficiencies cause children to have slower rates of growth. This issue seems more important in children over two years. Because in children under two years of age, maternal breastfeeding, could provide the children`s need for zinc [9]. In addition, the body store of zinc from in-utero development could partly compensate for the body’s need for zinc [10].

In this context, the World Health Organization (WHO) and the United Nations Children’s Fund are presently advocating for the use of a micronutrient powder formulation that contains 4.1-5 mg of zinc together with 14 other micronutrients [11]. In terms of zinc supplementation, some meta-analysis studies were conducted to assess its effect on the growth of children. Brown et al., systematically reviewed the effect of zinc supplementation in prepubertal children [12]. Imdad et al. assessed the effect of zinc supplementation on the growth pattern of under five-year-old children in developing countries [13]. Liu et al. reviewed the effect of zinc supplementation in children with growth retardation. Different original publications also assessed the effect of zinc supplementation on the growth pattern of healthy children and provided mixed results. To the best of our knowledge, there is no systematic review study that summarizes the result of these studies. So, the current meta-analysis was carried out to compile a summary of the evidence that is currently available on the influence of zinc supplementation on anthropometric parameters in healthy children.

## Materials and methods

The Preferred Reporting Items for Systematic Reviews and Meta-Analyses (PRISMA) procedure served as the basis for the planning, execution, and reporting of this work [14].

### Search strategy

Online databases including Scopus, Web of Science, and PubMed were searched to find all the relevant clinical trials up to November 2021. The search strategy for each database is provided in Table S1. The following search terms were used: Population: children; Intervention: Zinc, outcome: Anthropometric Measurements. In addition, we conducted a hand search of the reference lists of the relevant papers and review papers to include any other studies that might be suitable.

### Inclusion criteria

Clinical trials were included in the present systematic review and meta-analysis if they fulfilled the following inclusion criteria: (a) were randomized with either parallel or crossover designs in healthy children over 2 years; (b) reported anthropometric indices before and after intervention in each group; (c) compared zinc supplementation with the placebo. Studies were excluded if they: (a) do not use zinc alone; (b) did not provide effects sizes on anthropometric factors before and after the trial; (c): Abstracts, conference proceedings or book chapters, and unpublished studies.

Two investigators (ED and FA) selected eligible articles separately by reading titles, abstracts, and whenever required the full text of the publications. Any disagreements in this regard were resolved through discussion with the third researcher (ZF).

### Data extraction

Data extraction was done by two researchers independently (VM and AS). The disagreements were settled through discussion and by agreeing with the assistance of a third reviewer (ZN). The following information was extracted: name of the first author, publication year, individuals’ characteristics (mean age), study design, sample size, type of zinc prescribed, the dosage of zinc, unit, and duration of intervention. The corrected mean changes and standard deviations of anthropometric measurements were taken during the study for both the intervention group and the control group, as well as the variables that were considered to be confounding.

### Risk of bias assessment

The Cochrane quality assessment tool was used for assessing the risk of bias [15]. Two reviewers independently assessed the risk of bias (VM and AS) and the risk of bias was divided into high risk, low risk, and unclear risk.

### Certainty assessment

The overall certainty of evidence across the studies was graded according to the Grading of Recommendations, Assessment, Development, and Evaluations (GRADE) guideline, and the evidence was divided into high, moderate, low, and extremely low categories [16].

### Statistical analysis

The overall effect sizes were calculated using the mean changes in anthropometric measurements and their respective standard deviations (SD). In studies that reported the SEM, the SD was determined using the following formula: SD = SEM × √n, in which the n is the sample size. The I^2^ statistic and Cochrane’s Q test were used to determine the presence of heterogeneity. Significant heterogeneity was determined if the I^2^ value was > 50% or P < 0.05 [17]. To find probable sources of heterogeneity, subgroup analyses were performed according to the predefined variables including gender, country region (Asian or Non-Asian), sample size, and duration of studies (> 24,<24). A sensitivity analysis was performed to determine whether or not the overall effect size was dependent on a specific study. The formal test developed by Begg and Egger looked into the possibility of publication bias. Stata, version 14 was utilized for the meta-analysis, and a *P-value < 0.05* was considered a significant level.

## Results

### Study selection

**Fig. 1:**
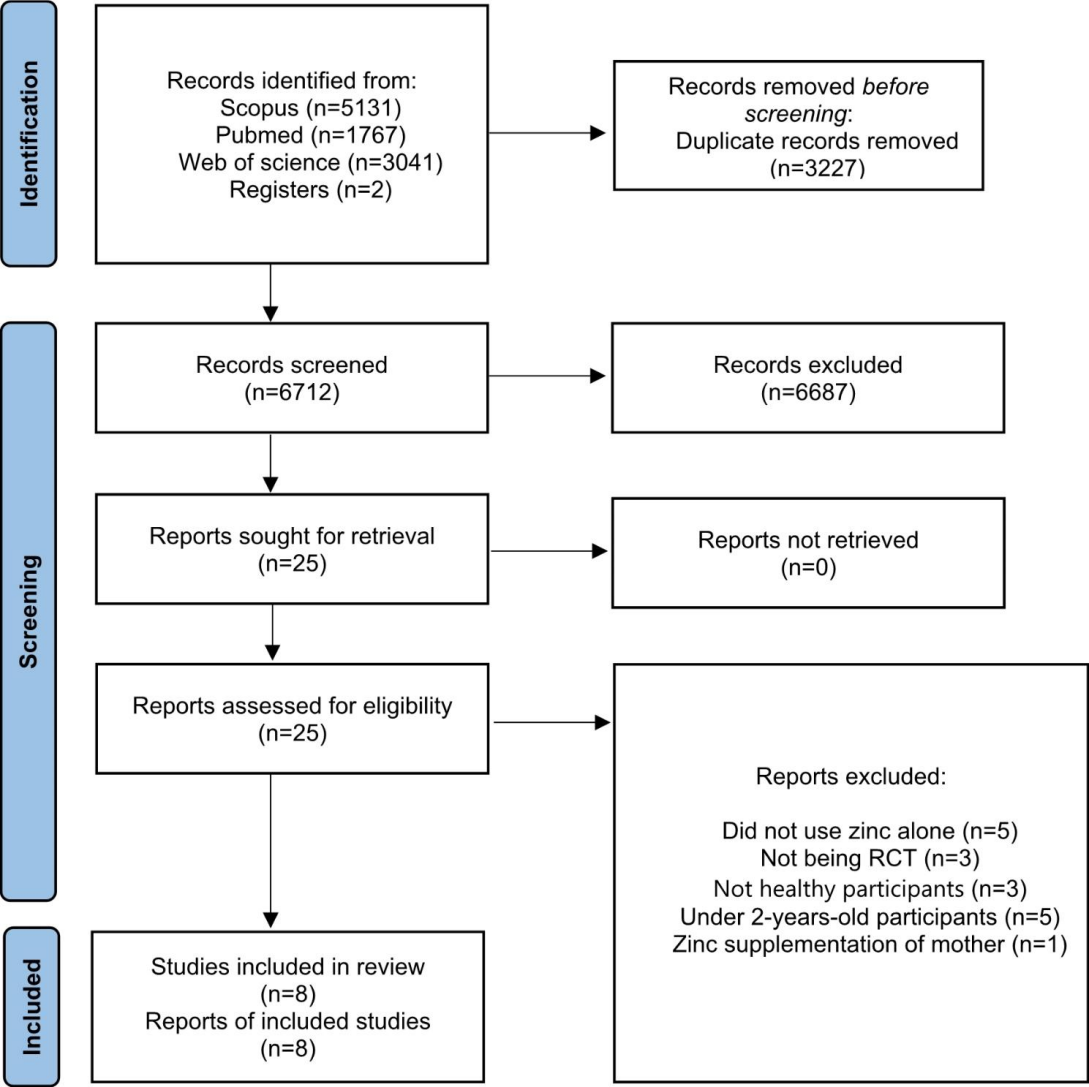
Flowchart of study selection for inclusion trials in the systematic review

As shown in Fig. 1, of 9939 publications that were retrieved in a systematic search, 25 articles remained for full-text evaluation. Additional 17 articles were excluded by full text for the following reasons: not being clinical trials [18–20], do not use zinc alone [21–25], non-healthy subjects [26–28], under 2 years [29–33], and zinc supplementation of mothers [34]. Finally, eight eligible RCTs remained for inclusion in the current systematic review and meta-analysis [35–42].

### Study characteristics

The characteristics of included RCTs are illustrated in Table 1. Two studies were performed on boys and two on girls and four on both genders. The sample size of included RCTs varied from 46 to 804 participants, resulting in a total sample size of 1586. The dosage of zinc supplements varied from 5 to 15 mg/day, and the duration of intervention ranged from 6 to 28 weeks.

**Table 1:** Characteristic of included studies in meta-analysis

studies	Study Design	Country	Sample size	Sample size		Trial Duration (Week)	Means Age(month)		Intervention		dose 15 mg/day
				**IG**	**CG**		**IG**	**CG**	**zinc**	**Control group**	
Ruz et al. 1997	RCT	USA	98	49	49	56	39/8	39/8	Zinc	placebo	10 mg/day
Kikafunda et al. 1998	RCT	Ugandan	153	78	75	32	55/5	56/1	Zinc sulfate	placebo	10 mg/day
Clark et al. 1999	RCT	Netherland	46	25	21	6	NR	NR	Zinc	placebo	15 mg/day
Ebrahimi et al. 2006	RCT	Iran/Yasuj	804	386	418	28	NR	NR	Zinc syrup	placebo	10 mg/day
Dehbozorgi et al. 2007	RCT	Iran/Shiraz	60	30	30	24	NR	NR	Zinc sulfate	placebo	8 mg/day
Kaseb et al. 2013	RCT	Iran/Yasuj	95	48	47	16	143/16	148/92	Zinc sulfate	placebo	5 mg/day
Vakili/girl et al.2015	RCT	Iran/Mashhad	100	50	50	24	94/5	95/64	Zinc sulfate	placebo	10 mg/day
Vakili/boy et al.2015	RCT	Iran/Mashhad	100	50	50	24	91/78	91/84	Zinc sulfate	placebo	10 mg/day
Sanguansak et al.2017	RCT	Thailand	130	66	64	24	106/8	105/6	Zinc bisglycinate	placebo	15 mg/day

Abbreviations: IG: intervention group; CG: control group; NR: not reported

### Risk of bias result

The result of the risk of bias is shown in Table 2. Only two studies [36, 37] could be considered high-quality studies, two RCTs [42, 43] were of moderate quality and others had low-quality.

**Table 2:** Risk of bias assessment for randomized clinical trials included in the current meta-analysis on the effect of zinc supplementation on Anthropometric factors in Healthy children over 2 years^1^

studies	Selection bias (random sequence generation)	Selection bias (allocation concealment)	Performance bias	Attrition bias	Detection bias	Reporting bias	Other sources of bias
Sedigheh ebrahimi et al. 2006	L	U	L	U	U	H	U
Joyce K Kikafunda et al. 1998	L	H	L	L	L	L	L
Peter J Clark et al. 1999	L	H	L	U	L	L	L
Sanguansak Rerksuppaphol et al. 2017	L	L	L	L	L	L	L
Manuel ruz et al. 1997	L	L	L	L	L	L	L
Rahim Vakili et al. 2015	L	U	L	L	L	L	L
Fatemeh Kaseb et al. 2013	L	L	H	L	H	L	L
P.dehbozorgi 2007	L	L	L	L	U	L	L

L: low risk H: high risk U: unclear

^1^Each study was assessed for risk of bias using the Cochrane Risk of Bias Assessment tool. Each domain was scored as “high risk” if it contained methodological flaws that may have affected the results, “low risk” if the flaw was deemed inconsequential, and “unclear risk” if information was insufficient to determine. If a study got “low risk” for all domains, it considered as a high-quality study with totally low risk of bias

### Grading of evidence

The GRADE protocol was used to assess the certainty of the evidence (Table 3). Accordingly, studies investigating the effects of zinc supplementation on weight and height were regarded as moderate quality due to the high heterogeneity between studies. Moreover, the effects of zinc supplementation on HAZ, and WAZ had moderate quality evidence. HAZ had low heterogeneity and WAZ has high heterogeneity.

**Table 3:** GRADE profile of the effect of zinc supplementation on Anthropometric factors in Healthy children over 2 years

Quality assessment						Summary of findings		Quality of evidence
Outcomes	Risk of bias	Inconsistency	Indirectness	Imprecision	Publication Bias	Number of intervention / control	WMD (95%CI)	
weight	No serious limitations	Very Serious limitations ^a^	No serious limitations	No serious limitations	No serious limitations	733/755	0.9 (0.27,1.52)	⊕⊕⊕◯ Moderate
height	No serious limitations	Very serious ^b^ limitations	No serious limitations	No serious limitations	No serious limitations	733/755	0.51 (0.06,0.97)	⊕⊕⊕◯ Moderate
WAZ	No serious limitations	serious limitations ^c^	No serious limitations	No serious limitations	No serious limitations	293/288	0.06 (-0.03,0.15)	⊕⊕⊕◯ Moderate
HAZ	No serious limitations	No serious limitations ^d^	No serious limitations	serious limitations	No serious limitations	293/288	0.07 (0.03,0.10)	⊕⊕⊕◯ Moderate

^WAZ: weight for age; HAZ: height for age^

^a^ The test for heterogeneity is significant, and the I^2^ is high, 93.9%

^b^ The test for heterogeneity is significant, and the I^2^ is high, 95.9%

^c^ The test for heterogeneity is significant, and the I^2^ is moderate, 72.4%

^d^ The test for heterogeneity is not significant, and the I^2^ is low, 33.1%

### Findings from the meta-analysis

Overall, eight RCTs were included in the meta-analysis. These trials had a total sample size of 1586 individuals aged two years and over.

### The effect of zinc supplementation on weight

**Fig. 2:**
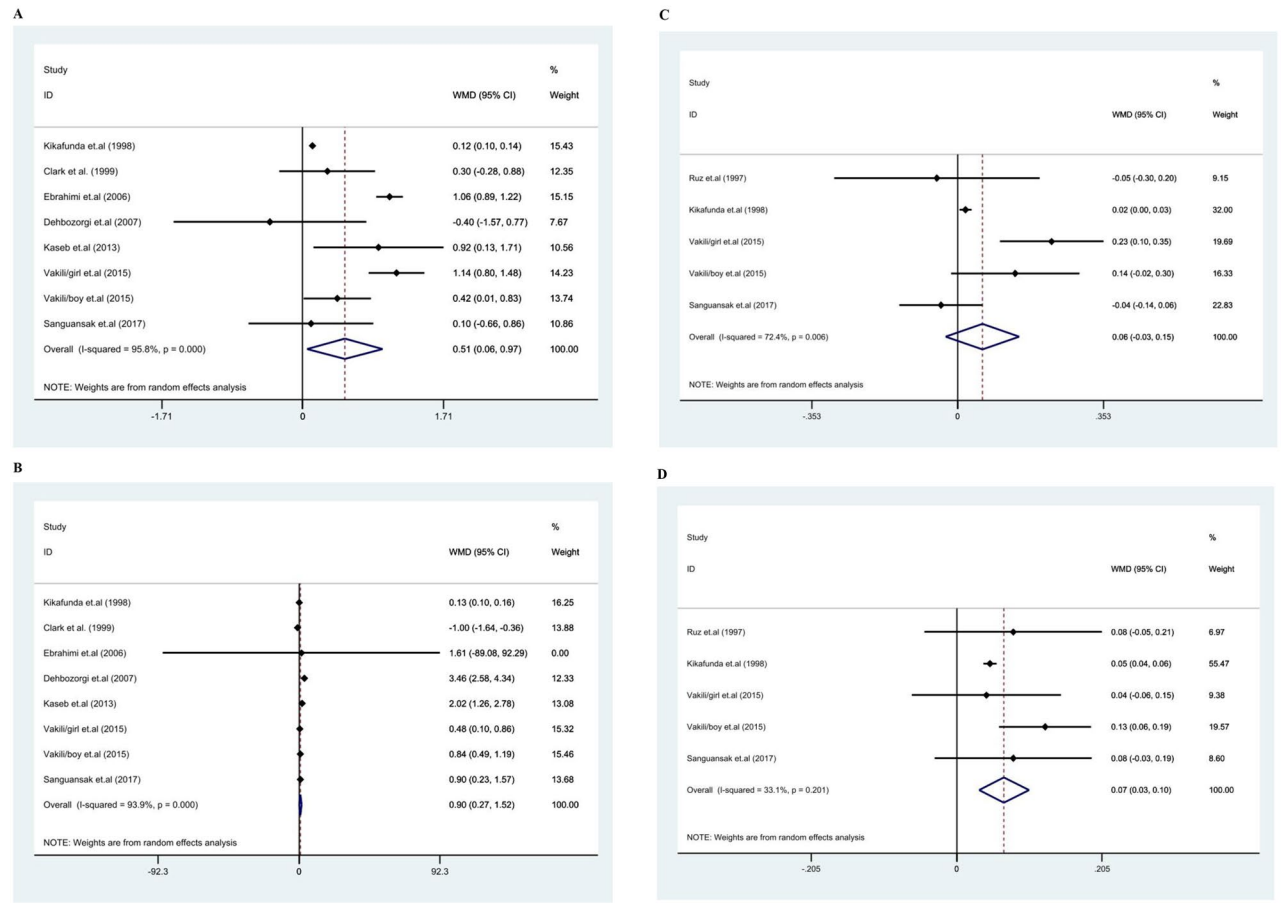
Forest plot for; **A**: weight, **B**: height, **C**: weight for age z-score (WAZ), **D**: height for age z-score (HAZ). WMD: weighted mean difference

The effects of zinc supplementation on weight were evaluated in seven research, including 1488 people [35, 36, 38–42]. Considering the presence of high heterogeneity between studies (*I*^2^ = 95.9, P < 0.001), the random-effect model was used. The result indicated that zinc supplementation had a significant impact on weight [(WMD): 0.51, 95% CI: (0.06, 0.97), *p* < *0.001*] (Fig. 2-B). The heterogeneity might be explained by the location of the study, duration of intervention, gender, and sample size. The subgroup analysis showed that the studies conducted in Asian countries (I^2^ = 0.0%, p = 0.545) and gender (boy, I^2^ = 40.4 p = 0.195) were the potential sources of heterogeneity (Table 4).

**Table 4:** Subgroup analyses the effect of zinc supplementation on Anthropometric factors in Healthy children over 2 years

	Number of studies	WMD (95% CI)	*I*^2^ (%)	P-between	P for heterogeneity
**The effect of zinc supplementation on height**					
**Location**					
Asian	2	0.13 (0.10, 0.16)	91.6	< 0.001	0.001
Non-Asian	6	0.99 (0.77, 1.22)	88.9		< 0.001
**Sample size**				< 0.001	
≥ 100	5	0.77 (0.55, 0.99)	94.9		< 0.001
< 100	3	0.13 (0.10, 0.16)	60.1		0.081
**gender**				< 0.001	
Girl	2	0.09 (-0.24, 0.42)	93.4		< 0.001
Boy	2	1.20 (0.08, 1.53)	96.6		< 0.001
both	4	0.13 (0.10, 0.17)	89.4		< 0.001
Duration (weeks)				< 0.001	
24<	2	0.13 (0.01, 0.16)	0		0.975
24≥	6	0.78 (0.57, 0.99)	93.6		< 0.001
**The effect of zinc supplementation on weight**					
**location**					
Asian	2	0.12 (0.10, 0.14)	0	< 0.001	0.545
Non-Asian	6	0.95 (0.82, 1.08)	74		< 0.001
Sample size				< 0.001	
≥ 100	5	0.73 (0.50, 0.95)	70.8		0.008
< 100	3	0.14 (0.12,0.16)	98.4		< 0.001
gender				< 0.001	
Girl	2	0.93 (0.63, 1.22)	83.3		0.015
Boy	2	0.33 (-0.06, 0.72)	40.4		0.195
both	4	0.14 (0.12, 0.16)	97.7		< 0.001
duration				< 0.001	
24<	2	0.14 (0.12, 0.16)	99.2		< 0.001
24≥	6	0.68 (0.46, 0.89)	49		0.007
**The effect of zinc supplementation on WAZ**					
location				0.09	
Asian	2	0.02 (0.00, 0.03)	0		0.588
Non-Asian	3	0.08 (0.01, 0.15)	82.4		0.003
Sample size				0.002	
≥ 100	3	0.16 (0.07, 0.25)	48.9		0.141
< 100	2	0.02 (0.00, 0.03)	22.3		0.257
gender				0.002	
Girl	1	0.23 (0.10, 0.35)			
Boy	1	0.14 (-0.02, 0.30)			
both	3	0.02 (00.0, 0.03)	0		0.456
duration				0.009	
24<	2	0.02 (0.00, 0.03)	0		0.05
24≥	3	0.08 (0.01, 0.15)	82.4		0.003
**The effect of zinc supplementation on HAZ**					
location					
Asian	2	0.05 (0.04, 0.06)	0	0.004	0.607
Non-Asian	3	0.10 (0.05, 0.15)	0		0.399
Sample size				0.050	
≥ 100	3	0.10 (0.05, 0.15)	0		0.403
< 100	2	0.05 (0.04, 0.06)	0		0.562
gender				0.006	
Girl	1	0.04 (0.06, 0.15)			
Boy	1	0.13 (0.06, 0.19)			
both	3	0.05 (0.04, 0.06)	0		0.742
duration				0.04	
24<	2	0.05 (0.04, 0.06)	0		0.607
24≥	3	0.10 (0.05, 0.15)	0		0.399

### The effect of zinc supplementation on height

The effects of zinc supplementation on height were evaluated in seven research, including 1488 people [35, 36, 38–42]. Considering the presence of high heterogeneity (*I*^2^ = 93.9, P < 0.001), the random effect model was used. The result showed that zinc supplementation had a significant effect on height [(WMD): 0.9, 95% CI: (0.27,1.52), *p* < *0.001*] (Fig. 2-A). As shown in Table 4, the heterogeneity might be explained by the location of the study, duration of intervention, gender, and sample size. The subgroup analysis showed that the intervention duration of ≥ 24 weeks (I^2^ = 0.0%, p = 0.975) was the potential source of heterogeneity. An intervention duration of greater than 24 weeks (WMD 0.78; 95% CI: 0.57 to 0.99, p < 0.000) significantly increased height compared with a shorter intervention duration (WMD: 0.13; 95% CI: 0.10 to 0.16, p = 0.975). Moreover, we found a significant increase in height with zinc supplementation in RCTs with a sample size of less than 100 (Table 4).

### The effect of zinc supplementation on WAZ

The effects of zinc supplementation on WAZ were evaluated in four studies (with five effect sizes), including 581 subjects [35–37, 40]. There was high heterogeneity between studies (*I*^2^ = 72.4, P = 0.006). The result of the random-effect model showed that zinc supplementation had a significant impact on WAZ [(WMD): 0.06, 95% CI: (-0.03, 0.15), *p-value = 0.18*] (Fig. 2-C). The heterogeneity might be explained by the duration of the intervention, gender, and sample size. The subgroup analysis showed that all subgroups were the potential sources of heterogeneity and we found a significant increase in WAZ with zinc supplementation with duration 24≥, and among non-Asian people (Table 4).

### The effect of zinc supplementation on HAZ

The effects of zinc supplementation on HAZ were evaluated in four research (with five effect sizes), including 581 people [35–37, 40]. There was no significant between-study heterogeneity (*I*^2^ = 33.1, P = 0.20). The result of the fixed-effect model indicated that zinc supplementation had no significant impact on HAZ [(WMD): 0.07, 95% CI: (0.03, 0.10), *p < 0.001*] (Fig. 2-D).

### Sensitivity analysis

The results of the sensitivity analysis revealed that the magnitude of the overall effect regarding the association between zinc supplementation and height, WAZ, and HAZ did not depend on a single study. However, in terms of weight, the total impact size depended on some studies [35–42]. As a result, when those studies were disregarded, a substantial positive correlation between zinc supplementation and weight was discovered.

### Publication bias

As can be seen on the funnel plot (Figure S1 A-D), there were no signs of publication bias in the studies that evaluated the effect of zinc supplementation on weight, height, WAZ, and HAZ.

### Meta-regression analysis

Meta-regression was used to investigate the potential linear association between dose and duration of intervention, with absolute changes in anthropometric indices. Accordingly, meta-regression analysis did not reveal any significant association between the dose of supplementation with weight changes (*p*_linearity = 0.54_), height (*p*_linearity = 0.09_), HAZ (*p*_linearity = 0.89_), WAZ (*p*_linearity = 0.43_) (Figure S2 A-D). Likewise, there was no significant association between the duration of intervention with weight changes (*p*_linearity = 0.92_), height (*p*_linearity = 0.51_), WAZ (*p*_linearity = 0.96_), HAZ (*p*_linearity = 0.31_) (Figure S3 A-D).

## Discussion

Child malnutrition is one of the main public health challenges worldwide and about 45% of under 5-year-old children’s death is attributed to undernutrition [44]. In this regard, zinc deficiency is considered one of the most important causes of morbidity [45]. So, different studies were conducted to assess the effect of zinc supplementation on child growth in healthy children over two years of age. The meta-analysis of these studies showed that zinc supplementation had a significant effect on healthy children’s weight and height. Previous meta-analysis in prepubertal children showed a significant, positive effect of zinc supplementation on height and weight [12]. In under-five-year-old children in developing countries, Imdad et al. demonstrated the significant effect of zinc supplementation on linear growth [13]. In children with growth retardation, Liu et al. showed a significant effect of zinc supplementation on height, and weight [10]. However, Ramakrishnan et al. did not show a positive effect of zinc supplementation on weight and height in under five-year-old children [46]. The differences between the results of the various meta-analysis may be partly related to the differences in the age group of children. It has been shown that zinc supplementation had a more positive effect on over two years of old children compared with infant. Moreover, the health status of children can affect the result. For example, Liu et al. conducted a meta-analysis study on children with growth retardation. However, in the present meta-analysis, we included the paper with healthy subjects.

The positive effect of zinc supplementation on weight and height may be related to the effect of zinc on the metabolism of growth hormones (GH). Zinc had a positive effect on the secretion and sensitivity of GH [47–49]. Moreover, zinc had an important role in the binding of GH to its receptors [50]. In addition, zinc regulates the expression of GH receptor and insulin-like growth factor 1 (IGF1) genes in the liver [51]. Besides, zinc supplementation increases the insulin-like growth factor-1, and insulin-like growth factor binding protein-3 in healthy children [52].

In the present study, we indicated the significant effect of zinc supplementation on HAZ but not WAZ of healthy children over two-year-old. Conversely, Liu et al. reported the significant effect of zinc supplementation on WAZ in children over two years. In the present systematic review, we included studies with healthy participants, however, Liu et al. conducted a systematic review on studies with children with retarded growth.

In subgroup analyses, we found that intervention duration, study location, gender, and sample size were significant sources of heterogeneity. A previous meta-analysis study indicated that a longer dose of zinc intervention resulted in a more effect on the risk factors [53]. In addition, gender differences have been observed in the association of zinc and other diseases [54]. Moreover, the higher prevalence of zinc deficiency in developing countries may justify the effect of study location on heterogeneity [55]. Besides, a previous study showed that studies with small sample sizes are more heterogeneous than large **ones** [56].

### Limitations

In the current meta-analysis, some potential limitations should be addressed. The publication bias could not be fully excluded. There was considerable heterogeneity between the included studies. Although we performed a subgroup meta-analysis for some factors, not all sources of statistical heterogeneity were recognized. In addition, not all included studies are methodologically sound. The potential methodological problems of studies were as follow selection bias, performance bias, detection bias, and reporting bias.

## Conclusion

Altogether, the results of this meta-analysis are in favor of the effect of zinc supplementation on weight, WAZ, and height in healthy under-two-year-old children. Considering the limitations of the included studies, these findings should be confirmed by more high-quality randomized clinical trials that focus on the long-term effects of zinc supplementation on anthropometric indices.

### Electronic supplementary material

Below is the link to the electronic supplementary material.

Supplementary Material 1

Supplementary Material 2

## Data Availability

The datasets generated and/or analyzed during the current study are not publicly available due to the institution’s policy, but are available from the corresponding author upon reasonable request.
