# Supplementary material for: The effect of zinc supplementation on anthropometric measurements in healthy children over two years: a systematic review and meta-analysis
Source: BMC Pediatr. 2023 Aug 23;23:414. doi: 10.1186/s12887-023-04249-x (PMC10464267; doi:10.1186/s12887-023-04249-x)
Supplement: Supplementary file 1 — Supplementary Material 1 [file 12887_2023_4249_MOESM1_ESM.docx]

**A**


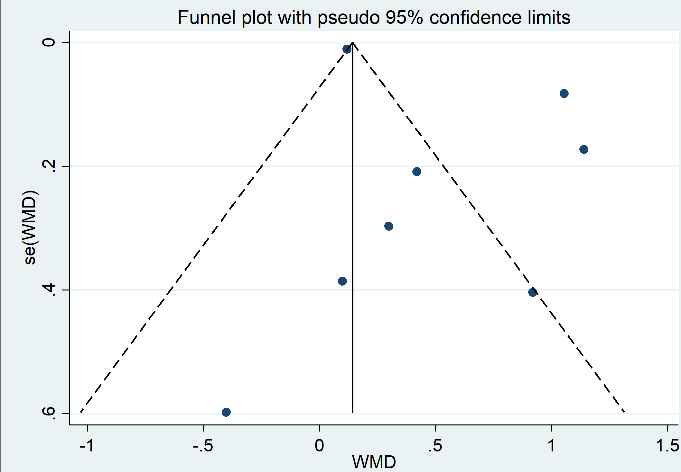


**B**


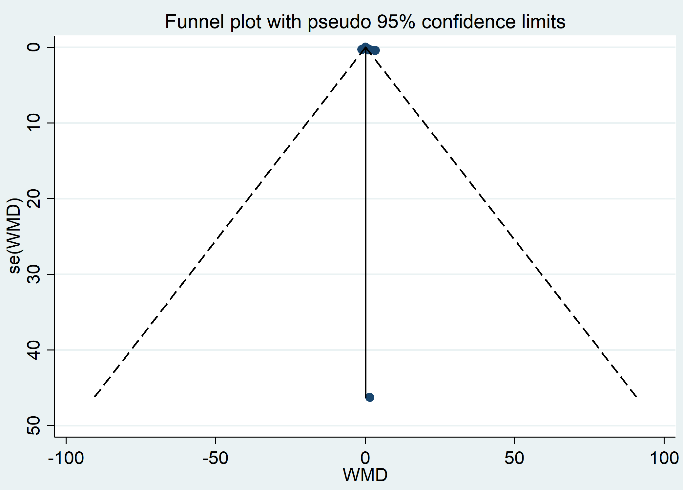


**C**


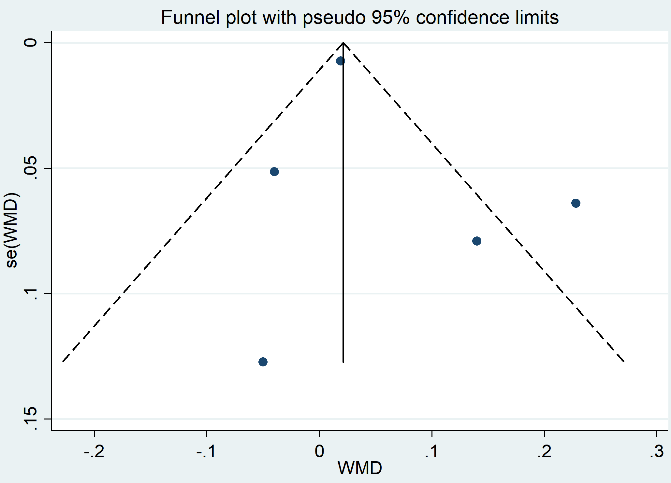


**D**


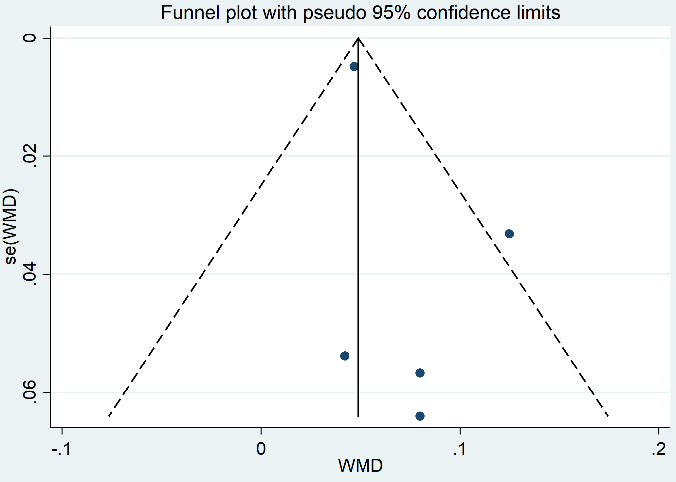


**Fig S1:** funnel plot for; A: weight, B: height, C: weight for age z-score (WAZ), D: height for age z-score (HAZ).

**A**

B

**C**

**D**

**Fig S2:** Meta-regression based on dose; A: height, B: weight, C: weight for age z-score (WAZ), D: height for age z-score (HAZ)

**A B**

**C D**

**Fig S3;** Meta-regression based on duration; A: height, B: weight, C: weight for age z-score (WAZ), D: height for age z-score (HAZ)
