# Supplementary material for: The effect of zinc supplementation on anthropometric measurements in healthy children over two years: a systematic review and meta-analysis
Source: BMC Pediatr. 2023 Aug 23;23:414. doi: 10.1186/s12887-023-04249-x (PMC10464267; doi:10.1186/s12887-023-04249-x)
Supplement: Supplementary file 2 — Supplementary Material 2 [file 12887_2023_4249_MOESM2_ESM.docx]

| PubMed | ((zinc[Title/Abstract] OR zn[Title/Abstract] OR "zinc sulfate"[Title/Abstract] OR "zinc gluconate"[Title/Abstract] OR "zinc supplementation"[Title/Abstract] OR "zinc picolinate"[Title/Abstract] OR "zinc citrate"[Title/Abstract] OR "zinc acetate"[Title/Abstract] OR "zinc monomethionine"[Title/Abstract]) AND (pediatric[Title/Abstract] OR teenage[Title/Abstract] OR adolescent[Title/Abstract] OR child[Title/Abstract] OR children[Title/Abstract])) AND (growth[Title/Abstract] OR "linear growth"[Title/Abstract] OR "Physical Growth"[Title/Abstract] OR "growth factor"[Title/Abstract] OR "Anthropometric Measurements"[Title/Abstract] OR "Anthropometric"[Title/Abstract] OR "Anthropometric factors"[Title/Abstract] OR "waist circumference"[Title/Abstract] OR "WC"[Title/Abstract] OR height[Title/Abstract] OR weight[Title/Abstract] OR "height for age"[Title/Abstract] OR haz[Title/Abstract] OR " length for age"[Title/Abstract] OR laz[Title/Abstract] OR length[Title/Abstract] OR "weight for age"[Title/Abstract] OR waz[Title/Abstract] OR stunting[Title/Abstract] OR wasting[Title/Abstract]) | 1767 |
| --- | --- | --- |
| Scopus | ( TITLE-ABS-KEY ( zinc  OR  zn  OR  "zinc sulfate"  OR  "zinc gluconate"  OR  "zinc supplementation"  OR  "zinc picolinate"  OR  "zinc citrate"  OR  "zinc acetate"  OR  "zinc monomethionine" )  AND  TITLE-ABS-KEY ( pediatric  OR  teenage  OR  adolescent  OR  child  OR  children )  AND  TITLE-ABS-KEY ( growth  OR  "linear growth"  OR  "Physical Growth"  OR  "growth factor"  OR  "Anthropometric Measurements"  OR  "Anthropometric"  OR  "Anthropometric factors"  OR  "waist circumference"  OR  "WC"  OR  height  OR  weight  OR  "height for age"  OR  haz  OR  " length for age"  OR  laz  OR  length  OR  "weight for age"  OR  waz  OR  stunting  OR  wasting ) ) | 5131 |
| Web of science | **TOPIC:** (zinc or zn or "zinc sulfate" or "zinc gluconate" or "zinc supplementation" or "zinc picolinate" or "zinc citrate" or "zinc acetate" or "zinc monomethionine") *AND* **TOPIC:** (pediatric or teenage or adolescent or child or children) *AND* **TOPIC:** (growth or "linear growth" or "Physical Growth" or "growth factor" or "Anthropometric Measurements" or "Anthropometric" or "Anthropometric factors" or "waist circumference" or "WC" or height or weight or "height for age" or haz or " length for age" or laz or length or "weight for age" or waz or stunting or wasting) | 3041 |

Table S1: search strategy
